# Known HIV status among adolescent women attending antenatal care services in Eswatini, Ethiopia and Mozambique

**DOI:** 10.1186/s12978-021-01090-2

**Published:** 2021-05-03

**Authors:** Joel Njah, Mary Ann Chiasson, William Reidy

**Affiliations:** 1 ICAP At Columbia University’s Mailman School of Public Health, 722 W. 168th St., New York, NY 10032 USA; 2 Department of Epidemiology, Mailman School of Public Health, Columbia University, New York, NY USA; 3 Division of Infectious Diseases, Columbia University Irving Medical Center, New York, NY USA

**Keywords:** Adolescent pregnancies, HIV testing results, ANC, PMTCT, Sub-saharan Africa

## Abstract

**Background:**

Antenatal care (ANC) clinics remain important entry points to HIV care for pregnant women living with HIV—including adolescents. Prior knowledge of their HIV status at ANC enrollment is crucial to providing services for prevention of mother-to-child transmission (PMTCT) of HIV. We examined known HIV status of pregnant adolescents and women in other age groups at ANC enrollment.

**Methods:**

A descriptive study of routinely reported PMTCT data from 419 facilities in Eswatini, Ethiopia, and Mozambique, from January through December 2018 was conducted. We assessed knowledge of HIV status by country for three age groups: adolescents aged 15–19 years, young women aged 20–24 years, and older women aged 25–49 years. We report HIV prevalence and proportions of known and newly diagnosed HIV infections in women, by age group and country. The data were summarized by frequencies and proportions, including their 95% confidence intervals.

**Results:**

Among the facilities examined, 52 (12.4%) were in Eswatini, 63 (15.0%) in Ethiopia, and 304 (72.6%) in Mozambique. Across three countries, 488,121 women attended a first ANC visit and 23,917 (4.9%) were HIV-positive. Adolescents constituted 22% of all ANC attendees, whereas young and older women represented 33% and 45%, respectively. HIV prevalence was lowest among adolescents than in other age groups in Eswatini (adolescents 11.9%, young 24.2% and older 47.3%), but comparable to young women in Ethiopia (adolescents 1.6%, young 1.6% and older 2.2%) and Mozambique (adolescents 2.5%, young 2.5% and older 5.8%), However, in each of the three countries, lower proportions of adolescents knew their HIV-positive status before ANC enrollment compared to other age groups: in Eswatini (adolescents 51.3%, young 59.9% and older 79.2%), in Ethiopia (adolescents 42.9%, young 63.7% and older 75.2%), and in Mozambique (adolescents 16.4%, young 33.2% and older 45.6%).

**Conclusion:**

Overall, adolescents made up nearly one-quarter of the women examined and had the least knowledge of their HIV status at ANC enrollment. Their HIV prevalence and known HIV-positive status varied widely across the countries examined. Adolescent-friendly sexual and reproductive health, and PMTCT services, before pregnancy, are needed to improve knowledge of HIV status and support pregnant adolescents and their infants.

**Plain English summary:**

Antenatal care (ANC) clinics are important for HIV testing of pregnant adolescents, who may not know their HIV-positive status at the first ANC visit. We describe data on pregnant adolescents and women in other age groups in ANC services to examine their prior HIV status at ANC enrollment across three African countries.

We examined data from 419 PMTCT sites in Eswatini, Ethiopia, and Mozambique from January-December 2018, to evaluate HIV testing results for adolescents, young and older women aged 15–19, 20–24 and 25–49 years, respectively. We report the number of women living with HIV and the proportions of known and newly identified women living with HIV, by age-group and country.

Across three countries, 488,121 women attended ANC and 23,917 (4.9%) were living with HIV. Adolescents constituted 22% of all ANC attendees, whereas young and older women represented 33% and 45%, respectively. HIV prevalence in each country compared to other age groups was lowest and varied among adolescents from 11.9% in Eswatini, to 1.6% in Ethiopia and to 2.5% in Mozambique. Also, fewer adolescents knew their HIV-positive status before ANC enrollment compared to young and older women from 51.3% in Eswatini, 42.9% in Ethiopia to only 16.4% in Mozambique.

Pregnant adolescents made up nearly one-quarter of all ANC attendees; a majority of them had no previously known HIV-positive status. Adolescent-friendly, sexual and reproductive health services, before pregnancy and in PMTCT services, are needed to support pregnant adolescents and their infants.

**Supplementary Information:**

The online version contains supplementary material available at 10.1186/s12978-021-01090-2.

## Background

Over the past decade, remarkable progress has been made towards eliminating perinatal transmission of HIV through the global scale-up of prevention of mother-to-child transmission (PMTCT) of HIV services, particularly in sub-Saharan Africa (SSA) [1]. However, the risk of acquiring HIV infection is disproportionately higher among adolescent girls who together with young women under age 25 account for 30% of new HIV infections, though they make up just 10% of the population [2, 3]. HIV/AIDS was reported as the second leading cause of death in South Africa in 2015 among adolescent girls and young women (AGYW) [4], and the trend in mortality is projected to increase among this age group [5]. In response, a global target of fewer than 100,000 new HIV infections among AGYW aged 15 to 24 years by 2020 was established [2]. Recent reports, however, indicate that this target has been missed because of the slow decline in new HIV infections among AGYW [6, 7]. The slow decline might be due to the increased vulnerability of adolescent girls to HIV infection that is compounded by high rates of adolescent pregnancies in SSA. To shed light on the high prevalence of adolescent pregnancies, a United Nations Population Fund (UNFPA) global review, found that more than 25% of young women, including adolescents, examined had been pregnant by age 18 [8]. Exceptionally high pregnancy rates were reported in the Eastern and Southern regions of Africa, in particular in Mozambique, where over 40% of adolescent girls had at least one pregnancy [8].

Given the high pregnancy rates and disproportionately high HIV incidence among adolescent girls in SSA, substantial numbers of adolescent girls living with HIV (ALHIV) will be in need of antenatal care (ANC) and PMTCT services. ANC services serve as critical entry points for HIV testing and linkage to PMTCT care [9]; however, previous data from Zimbabwe have shown that only 3.1% of adolescents had a knowledge of their HIV status at ANC enrollment [10]. This is important because knowledge of HIV status prior to or during a first ANC visit may impact the quality and long-term engagement of adolescents in PMTCT services. In South Africa for example, a high LTFU rate of 57.5% was observed among newly identified women living with HIV (WLHIV), initiating antiretroviral therapy (ART) at a first ANC visit in a major urban clinic [11]. Looking specifically at adolescents initiating ART in three Eastern African countries, a recent publication found that pregnant adolescents aged 15–19 years had a higher risk of LTFU from HIV care when compared to their non-pregnant female counterparts [12].

Collectively, the extant literature reveals gaps in knowledge of HIV status among pregnant adolescents before ANC enrollment. We sought to analyze routine facility-level data from PMTCT sites in three SSA countries to understand gaps in HIV status knowledge among pregnant women of different age groups at a first ANC visit.

## Methods

### Study setting and design

This analysis used routine reporting data from PMTCT facilities in Eswatini, Ethiopia and Mozambique supported by ICAP at Columbia University through funding from the US President’s Emergency Plan for AIDS Relief (PEPFAR). Facility-level, age-disaggregated PMTCT program data were collected from January through December 2018. Of a total of 419 ICAP-supported facilities, 52 sites are in Eswatini; a Southern African country with four administrative regions, including Manzini where all the 52 sites are located. This landlocked country has a population of about 1.3 million inhabitants with an HIV prevalence of 27%; the highest in the world [13, 14]. Sixty three (63) of the ICAP-supported sites are located in four of the 9 administrative regions of Ethiopia, which are Afar, Somali, Benishagul-Gumuz and Gambella. Ethiopia is an Eastern African country with over 114 million inhabitants and a generalized HIV prevalence of 0.9% [15]. The bulk of the data for this analysis were obtained from 304 ICAP-supported sites in Mozambique. Mozambique is a Southern African country with more than 29 million inhabitants distributed in ten administrative provinces with a national HIV prevalence of 12.4%. These ICAP-supported sites are located in the two most populous provinces of Nampula and Zambezia. Nampula has an HIV prevalence of 6.5% compared to 14.5% in Zambezia.

### Data source and outcome variables

Data for pregnant women receiving PMTCT services were routinely collected by facility health personnel and aggregated at the facility level for reporting to national ministries of health and to Centers for Disease Control and Prevention (CDC)/PEPFAR. The data on numbers of first ANC attendees, previous knowledge of HIV-positive status and HIV-positive diagnosis in ANC were examined for three age groups that included adolescents aged 15–19 years, young women aged 20–24 years, and older women aged 25–49 years. Younger adolescents 10–14 years old were excluded from the analysis because of lack of disaggregated data on ‘known’ or ‘newly’ diagnosed as living with HIV that were needed to calculate the proportions. The outcome variables were the age-stratified prevalence of WLHIV, comprising those with known HIV-positive status prior to ANC enrollment plus those who newly diagnosed as living with HIV at ANC enrollment among all ANC clients; and the proportion of women previously known to be living with HIV among all women diagnosed as living with HIV were assessed across three countries [16]. Across ICAP-supported facilities, known HIV-positive status was confirmed for clients not on antiretroviral therapy (ART) and for those newly diagnosed as living with HIV, positive HIV-status was established by conducting two serial rapid HIV diagnostic tests according to WHO guidelines [17]. A commonly used first rapid test is the Abbott Determine™ HIV-1/2 Ag/Ab strips (Abbott Laboratories, Abbott Park, Illinois, USA), but the choice of the serial rapid tests varied by country [18].

### Data analysis

Our findings were described and summarized by frequencies and proportions, including their 95% confidence intervals (CI) and graphs of proportions by age groups for each country. We also conducted a Kruskal Wallis test to compare the mean ranks of the prevalence among women with known HIV status by country and age group. The age-disaggregated data were downloaded from the ICAP central database as excel sheets and imported into STATA version 15.0 (StataCorp, College Station, TX) for analysis.

## Results

### Study health facilities

Overall, 419 ICAP-supported PMTCT facilities were examined, including 52 (12.4%) in the Manzini region of Eswatini, 63 (15.0%) in Afar, Benishangul-Gumuz, Gambella and Somali regions of Ethiopia, and 304 (72.6%) in Nampula and Zambezia provinces of Mozambique.

(Fig. 1).

**Fig. 1:**
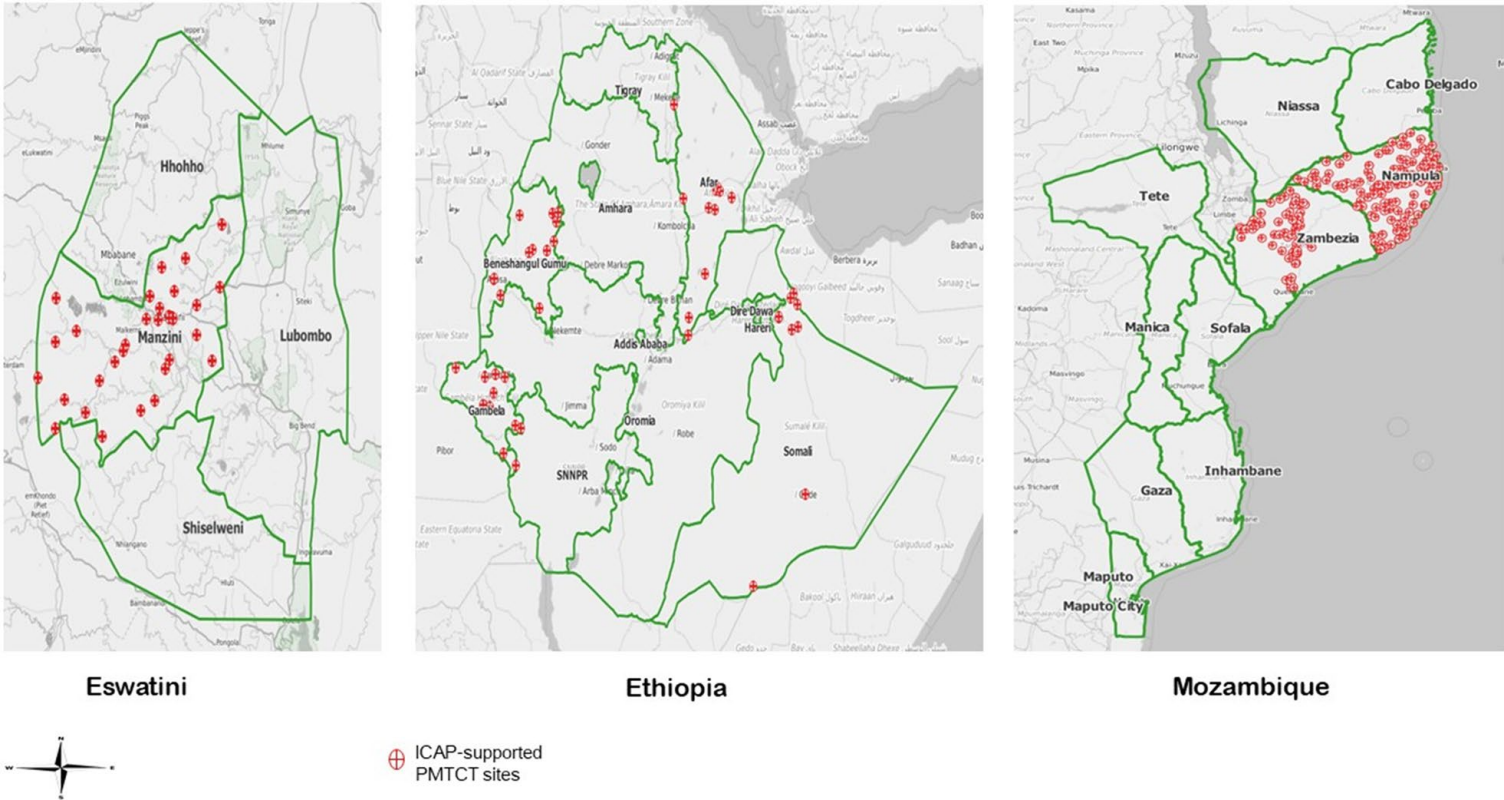
The geospatial maps of ICAP-supported sites in Eswatini, Ethiopia, and Mozambique. Not mapped to scale

### Study population

A total of 488,121 pregnant women attended a first ANC visit between January and December 2018 across the 419 health facilities in the three countries. Of these, 483,246 (99.0%) women aged 15–49 years were included in the analysis; 4,875 women who were either younger than 15 years or older than 49 years were excluded from the analysis. Among those included in the analysis, 23,917 (4.9%) were living with HIV (Table 1).

**Table 1:** Number of new ANC clients, prevalence and proportions of women with previously known HIV-positive status at ANC enrollment by age group and country in 2018

	Age group (years)	Eswatini, N = 8509 (%)	Ethiopia, N = 43,893 (%)	Mozambique, N = 435,719 (%)
ANC clients by age group	15–19	1313	6524	98,694
	20–24	2497	15,081	141,632
	25–49	4678	22,254	190,575
All HIV-positive women among new ANC clients^a^		2975 (35.0)	822 (1.9)	20,120 (4.6)
All HIV-positive women among new ANC clients by age group^a,b^	15–19	156 (11.9)	105 (1.6)	2490 (2.5)
	20–24	604 (24.2)	234 (1.6)	6497 (2.5)
	25–49	2212 (47.3)	483 (2.2)	11,100 (5.8)
Women with previously known HIV status among all HIV-positive women by age group^c^	15–19	80 (51.3)	45 (42.9)	408 (16.4)
	20–24	362 (59.9)	149 (63.7)	2156 (33.2)
	25–49	1751 (79.2)	363 (75.2)	5065 (45.6)

*ANC* antenatal care

^a^Overall prevalence of HIV-positive pregnant women

^b^Prevalence of HIV-positive pregnant women by age group

^c^Proportion of pregnant women with previously known HIV-positive status before ANC enrollment by age group

### HIV prevalence among all women attending a first ANC visit

**Fig. 2:**
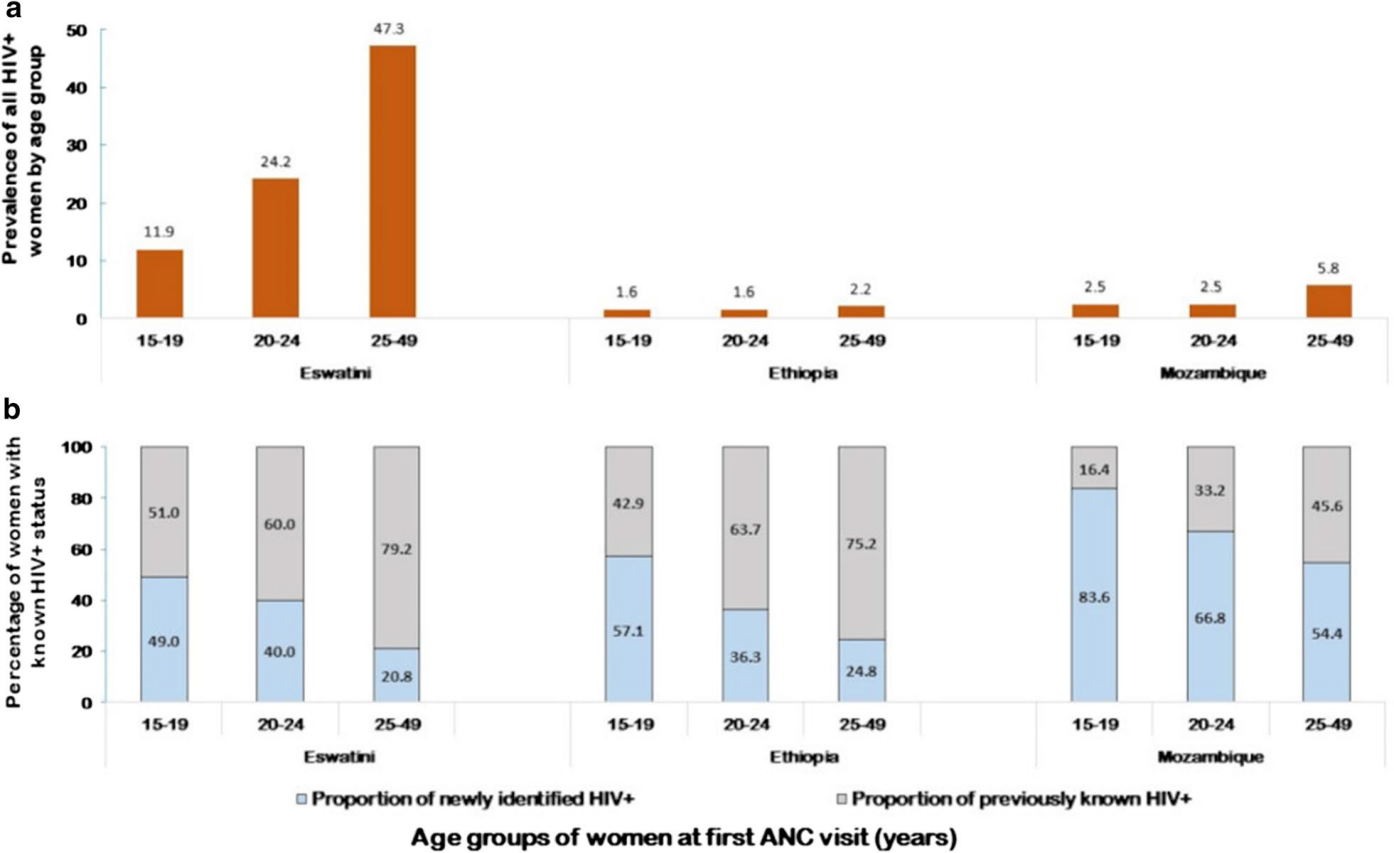
The prevalence of HIV-positive women and proportion of women with previous knowledge of their HIV-positive status at ANC enrollment bay age group. **a** Prevalence of all HIV-positive women by age group among first visit ANC attendees. **b** Percentage of women with prior knowledge of their HIV-positive status and those newly tested HIV-positive among all HIV-positive women at ANC enrollment

Overall, across three countries, adolescents constituted 22.0% (106,531/483,248) of all women seeking a first ANC visit, while 32.9% (159,210/483,248) and 45.0% (217,507/483,248) of them were young and older women, respectively. By country, HIV prevalence was lowest among adolescents in Eswatini (adolescents 11.9% [95% CI: 10.2–13.6], young 24.2% [95% CI: 20.5–27.9], and older 47.3% [95% CI: 45.8–48.9]), but similar to that of young women in Ethiopia (adolescents 1.6% [95% CI: 1.0–2.3], young 1.6% [95% CI: 1.0–2.1] and older 2.2% [95% CI: 1.4–3.0]), and Mozambique (adolescents 2.5% [95% CI: 2.2–2.9], young 2.5% [95% CI: 3.6–5.5], and older 5.8% [95% CI: 5.0–6.5]) (Fig. 2a). The prevalence of HIV-positive women and proportion of women with previous knowledge of their HIV-positive status at ANC enrollment bay age group. a Prevalence of all HIV-positive women by age group among first visit ANC attendees. b Percentage of women with prior knowledge of their HIV-positive status and those newly tested HIV-positive among all HIV-positive women at ANC enrollment. The prevalence of HIV-positive women and proportion of women with previous knowledge of their HIV-positive status at ANC enrollment bay age group. a Prevalence of all HIV-positive women by age group among first visit ANC attendees. b Percentage of women with prior knowledge of their HIV-positive status and those newly tested HIV-positive among all HIV-positive women at ANC enrollment. The prevalence of HIV-positive women and proportion of women with previous knowledge of their HIV-positive status at ANC enrollment bay age group. a Prevalence of all HIV-positive women by age group among first visit ANC attendees. b Percentage of women with prior knowledge of their HIV-positive status and those newly tested HIV-positive among all HIV-positive women at ANC enrollment.

### Proportions of women known to be living with HIV at ANC enrollment

Across three countries, adolescent women had the lowest proportion of women known to be living with HIV before ANC enrollment at 11.5%, whereas young and older women represented 30.7% and 57.8%, respectively. The proportion of all WLHIV with known HIV positive status, as opposed to those newly diagnosed at ANC enrollment, across all age groups was highest in Eswatini at 73.7%, followed by 67.8% in Ethiopia and 37.9% in Mozambique. Across three countries, smaller proportions of adolescent pregnant women knew their HIV-positive status before ANC enrollment; in Eswatini (adolescents 51.3% [95% CI: 27.8–77.6], young 59.9% [95% CI: 45.0–76.7] and older 79.2% [95% CI: 75.0–83.2]), Ethiopia (adolescents 42.9% [95% CI: 27.9–60.2], young 63.7% [95% CI: 47.3–80.0] and older 75.2% [95% CI: 69.0–82.6]), and Mozambique (adolescents 16.4% [95% CI: 11.4–21.1], young 33.2% [95% CI: 26.2–39.0] and older 45.6% [95% CI: 40.1–50.5]) (Fig. 2b).

Comparing the HIV prevalence among women with known HIV status by countries and age groups, there was a statistically significant increase among adolescent women between: Eswatini and Mozambique (p-value = 0.004), Ethiopia and Mozambique (p-value = 0.02), but not between Eswatini and Ethiopia (p-value = 0.278). A similar trend was observed among young women, Eswatini and Mozambique (p-value = 0.016), Ethiopia and Mozambique (p-value = 0.005), but not between Eswatini and Ethiopia (p-value = 0.347), and among older women, Eswatini and Mozambique (p-value = 0.004), Ethiopia and Mozambique (p-value = 0.02), but not between Eswatini and Ethiopia (p-value = 0.278).

## Discussion

In this analysis, we found that nearly a quarter (22.0%) of the women seeking ANC services were adolescent aged 15–19 years and that the proportions of adolescent women with known HIV-positive status before ANC enrollment varied considerably by country, ranging from 51% in Eswatini to 43% in Ethiopia to only 16% in Mozambique. We also found that lower proportions of pregnant adolescent knew their HIV-positive status before ANC enrollment compared to women in the young and older age groups across three countries. This report underscores the need to promote and expand adolescent-friendly HIV testing services to increase the HIV status knowledge of adolescent girls prior to a first ANC visit [19, 20].

Our findings are consistent with previous work by investigators in South Africa, who showed that adolescent women below 20 years of age were two times more likely than older women not to know their HIV-positive status before attending ANC services [21]. Musarandenga et al., assessing the uptake of PMTCT services among women attending ANC services in Zimbabwe, found that only 3% of adolescent women had knowledge of their HIV status at ANC enrollment [10]. Similarly, other studies in South Africa [22], and Kenya [20], showed that 75% and 70% of pregnant adolescents did not know their HIV status before ANC enrollment, respectively.

The existing body of literature, points to a relatively low overall HIV testing coverage among non-pregnant adolescents [10, 23]. For example, only 19% of girls and 14% of boys aged 15–19 in the Eastern and Southern Africa region tested for HIV and got their results in 2018 [24]. Reasons for this low uptake in HIV testing among adolescents according to a recent study include: low perception of HIV infection risk, fear of HIV-related stigma, and discomfort with finding out they are HIV-positive [4]. Testing for HIV and active linkage to care and treatment following a positive test result are critical steps in the HIV care continuum for adolescents. For instance, less than half of the adolescents who tested positive for HIV initiated ART in South Africa according to a recent national laboratory study [25]. There is therefore, a need to identify effective interventions that can improve HIV testing among adolescents before they become pregnant. The DREAMS (Determined, Resilient, Empowered, AIDS-free, and Safe) initiative is one of such adolescent-targeted programs that need to be expanded to priority and key adolescent girls to improve their HIV status knowledge before pregnancy [26–28].

Pregnant adolescents face additional challenges and have been shown to be more vulnerable to LTFU after receiving a positive HIV test result during ANC enrollment [29]. While data are limited on the reasons for this increase vulnerability, it may be related to the extra burden of processing new information on testing positive for HIV and initiating ART on the same day, on top of the routine ANC demands. However, adolescent-friendly services are limited in existing PMTCT services. Targeted-adolescent and youth-friendly services have been suggested as solutions to the challenges faced by adolescents in lifelong HIV care and treatment services [23, 30–32]. There is, therefore, an urgent need for innovative service delivery models for pregnant adolescents in ANC and PMTCT services to improve their health outcomes [33, 34].

### Limitations

The data used in our analyses have some limitations. The facilities we examined include only a small subset of all ANC facilities within the three countries; thus, our findings are not necessarily generalizable to the respective countries. In addition, other than age, the lack of data on hand on patient-level and facility-level characteristics, reduced our ability to assess associations between potential risk factors of poor knowledge of HIV status and PMTCT health outcomes. Future research would be needed to address this limitation. Despite these limitations, the current study provides additional evidence on gaps to fill on improving knowledge of HIV status among adolescent mothers before ANC enrollment.

## Conclusion

Our analysis showed that nearly one-quarter of all women living with HIV attending PMTCT services were adolescents, and that a substantial number of them were newly diagnosed with HIV infection at ANC enrollment. Also, lower proportions of adolescents, than women in other age groups, knew their HIV status. Adolescent-friendly sexual and reproductive health, and PMTCT services, before pregnancy, are needed to improve knowledge of HIV status and support these adolescent mothers and their infants.

## Supplementary Information

**Additional file 1:** Dataset.

## Data Availability

The dataset supporting the conclusions of this article is included within the article under Additional files (see Additional file 1).

## Abbreviations

MTCT: Mother-to-child transmission of HIV
PMTCT: Prevention of mother-to-child transmission of HIV
ANC: Antenatal care
SSA: Sub-Saharan Africa
AGYW: Adolescent girls and young women
LTFU: Loss to follow-up
DREAMS: Determined, Resilient, Empowered, AIDS-free, Mentored, and Safe.
WLHIV: Women living with HIV
ALHIV: Adolescents living with HIV
